# Genetic Factors Influencing Drug-Induced Liver Injury: Do They Have a Role in Prevention and Diagnosis?

**DOI:** 10.1007/s11901-017-0363-9

**Published:** 2017-08-07

**Authors:** Kathleen E. Clare, Michael H. Miller, John F. Dillon

**Affiliations:** The GUT Group, Division of Molecular and Clinical Medicine, School of Medicine, Ninewells Hospital and Medical School, University of Dundee, James Arnott Drive, Dundee, DD1 9SY UK

**Keywords:** Drug-induced liver injury, Genetic association, Biomarker, Predisposition: Amoxicillin/clavulanate, Flucloxacillin

## Abstract

**Purpose of Review:**

The pathogenesis of DILI is currently unknown; however, research has shown strong genetic associations with some DILIs. This paper describes the variant alleles uncovered by GWAS and discusses their potential role as susceptibility biomarkers.

**Recent Findings:**

An association with HLADRB1*15:01 and amoxicillin/clavulanate DILI has been shown by a number of research groups. The presence of the HLA-B*57:01 allele has been associated with an 81-fold increased risk of flucloxacillin DILI. The HLA-B*35:02 allele has significant association with minocycline DILI.

**Summary:**

With the exception of abacavir for HIV therapy, no other prospective genetic screening tests have met the threshold for clinical application. This is largely because DILI incidence is too low to warrant the cost and effort associated with testing. Perhaps, with the development of personalised medicine, a panel of genes for disease susceptibility, drug efficacy and adverse reactions could be tested once off. This would change the cost-effectiveness paradigm, personalise healthcare and reduce DILI risk by avoiding medications in patients with specific HLA alleles.

Drug-induced liver injury (DILI) is the leading cause of acute liver failure in the UK and USA [1, 2]. It accounts for approximately 50% of all cases and is associated with high morbidity and mortality. One study found that patients with DILI-associated jaundice had an 11.7% chance of progressing to fulminant hepatic failure resulting in either death or transplantation [3]. Although, paradoxically, this condition is rare, occurring in only 1 in every 10,000 patients receiving DILI-associated drugs, these medications are used frequently in clinical practice [4]; therefore, healthcare impacts on patient safety and cost are significant, over and above this most DILI does not cause overt liver failure but leads to patient morbidity and consumption of health care resource.

DILI arises from an interplay between pharmacodynamic drug properties acting with specific genetic/non-genetic host factors; reactions which are not predictable from drug dosing. The idiosyncratic and diverse disease presentation in DILI makes research challenging as the mechanistic understanding of this condition is still limited [5]. DILI is typically a clinical diagnosis of exclusion and management includes prompt cessation of the offending drug with supportive and symptomatic care [6]. Limitations in DILI prediction, diagnosis and clinical outcome have driven the growing interest for the development of new specific and sensitive biomarkers.

Although genetic factors have been associated with DILI since the 1980s, the development of genome-wide association studies (GWAS) has enabled the detection and confirmation of specific genetic risk factors [4]. Using this technique, a number of genetic links with DILI have been revealed. The data from these studies may provide support for the use of genetic variants as a biomarker for susceptibility to DILI for a given medication. This type of test would be classed as a susceptibility or risk biomarker. Before the clinical implementation of such screening, a biomarker must be qualified. A framework for this already exists.

In general terms, stages in biomarker development include the discovery of association, validation for its intended context of use and optimization for population use and clinical application [7]. The association between genetic factors and DILI have existed for decades; this addresses the first stage in biomarker development of initial discovery. With regard to the secondary stage validation, there are a select number of medications with various studies to support their genetic association with DILI [8–10]. This research continues for various other DILI-related drugs. The next step is to investigate the benefit of prospective pharmacogenetic testing in clinical situations to optimise drug choice or patient management. The study design may follow a similar structure to that of PREDICT-1, a first of its kind trial (discussed below) [11]. Once a marker and its method of analysis have gone through biological and technical development, the clinical effectiveness of its use must be assessed [7]. The regulatory body will require a biomarker to show clinical validity, utility, cost-effectiveness and safety before it can be used in medical practice [12].

Using HIV therapy as an exemplar, patients with the human leukocyte antigen (HLA)B*5701 allele are at an increased risk of developing a hypersensitivity reaction to the nucleoside reverse transcriptase inhibitor abacavir [13]. Trials were developed to validate the use of the HLA-B*5701 allele as a biomarker for abacavir hypersensitivity. Following on from this, PREDICT-1 was the first fully powered, randomised and double-blinded study designed to determine the effectiveness of prospective pharmacogenetic screening of HLA-B*5701 in preventing hypersensitivity reactions [11]. The results showed that prospective HLA-B*5701 testing reduced hypersensitivity reactions from 2.7% in the control group to 0% in the prescreened group. Research has proven that this screening test is cost effective [14]. One reason for this is the high positive predictive value of the test [15, 16]. The design of this trial may have utility in exploring the benefits of prospective pharmacogenetics testing for other key pharmacological agents with unfavourable and costly side effects such as those associated with DILI. This being said, the low positive predictive value of genetic associations in DILI, which will be discussed in the following sections, make prospective genetic testing less likely to be cost effective.

## Amoxicillin and Clavulanate-Associated DILI

Amoxicillin/clavulanate is the single most common causal agent of idiosyncratic DILI reported in registries, 10–13% of DILI-related hospitalisations [8, 17]. Amoxicillin is a commonly used antimicrobial which treats a variety of infections and has a relatively low side effect profile. The incidence of DILI with this agent alone is 0.3 per 10,000 prescriptions, much lower than the reports from amoxillicin/clavulanate use which is quantified as 1.7 per 10,000 prescriptions [18, 19]. The injury is thought to be from clavulanate, it is idiosyncratic but increases with age, repeated exposure, male sex and longer duration of treatment [18, 19]. The pattern of damage is variable. A predominantly hepatocellular injury tends to occur in younger individuals with shorter durations of treatment whilst cholestatic and mixed damage, the most common form, occurs more frequently in older patients with longer treatment regimens [20, 21].

The HLA genetic association between amoxicillin/clavulanate and DILI was first established by Hautekeete et al. in 1999. This group reported that the frequency of a HLA haplotype specifically antigen–D Related (DR) DRB1*15:01-DRB5*01:01-DQB1*06:02 was significantly increased in a European population of patients with amoxicillin/clavulanate-induced DILI compared to control subjects (57.1% compared to 11.1%) [22]. This association between HLADRB1*15:01 and amoxicillin/clavulanate-induced DILI has been replicated by a number of studies, [22, 23]. Donaldson et al. conducted a large national study and found the frequency of *DRB11501* in DILI patients was significantly higher than the population control: 53 and 30%, respectively [8]. The study also enrolled a ‘treatment control’ group of patients who had been exposed to amoxicillin/clavulanate with no reported side effects. Within this treatment control group, 30% of patients had *DRB11501.* This highlights the complex nature of genetic studies; not all patients with the gene or single nucleotide polymorphism develop the disease and conversely not all patients with the disease have the associated genetics. One study in a Spanish cohort failed to replicate this association [24].

A study which followed on from this identified two novel genetic factors associated with amoxicillin/clavulunate-induced DILI: HLA-A*02:01 [odds ratio 2.2 (95% CI 1.6–3.2)] in all patients and HLA-B*18:01 [odds ratio 4.0 (95% CI 1.5–11)] in Spanish patients alone. These results suggest that this reaction which is associated with MHC class I molecules may be dependent on ethnicity. A similar finding was reported regarding to HLA-B*15:02 and carbamazepine-induced severe skin reactions as this association was only applicable in certain Asian cohorts [25].

One study has linked specific haplotypes with specific clinical forms of DILI, cholestatic DILI has been associated with the DRB11501/DQB10602 haplotype whilst A3002/HLA B1801 is has been linked with hepatocellular DILI [26]. Another study has highlighted that patients who have double-null heterozygosity for glutathione *S*-transferase (GSTT1/GSTM1), an enzyme which detoxifies electrophilic compounds, are at an increased risk of amoxicillin/clavulanate-induced DILI [27].

In 2011, Lucena et al. conducted the most powerful and comprehensive study into the genetic risk factors associated with amoxicillin/clavulanate DILI. This was achieved through GWAS which looked at a large number of single nucleotide polymorphism markers from 201 Caucasian European and US patients with amoxicillin/clavulanate DILI. A large number of control patients, 532, were matched for genetic background. The results from this study not only confirmed the previous associations of HLA DRB1*15:01 and DQB1*0602 with amoxicillin/clavulanate DILI but also identified additional HLA risk factors with an apparent statistical interaction between two HLA alleles. High resolution HLA genotyping with 177 cases and 219 controls confirmed associations of HLA-A*0201 (*P* = 2 × 10^−6^) and HLA-DQB1*0602 (*P* = 5 × 10–6) and their interaction (*P* = 0.005) [28]. Additional population dependant effects were observed in HLA alleles with minimal significance.

Assuming the prevalence of amoxicillin/clavulanate-associated DILI is 0.014%, the best positive predictive value generated from this study is 0.1% for Northwestern Europeans based on the presence of both A*0201 and DQB1*0602 (with a frequency of 41% in cases). This value of 0.1% signifies that HLA genotyping will not be an effective method to prospectively identify those patients at risk of amoxicillin/clavulanate DILI; however, the data suggests that HLA genotyping may be of value in strengthening the diagnosis of amoxicillin/clavulanate DILI in view of the high negative predictive values noted. Based on the absence of A*0201 in Northwestern Europeans (74% carriage frequency in cases), the best negative predicative value is 0.006%. This value was 0.007% for the Spanish cohort based on the absence of *rs2523822/C* (74% carriage frequency in cases) [28].

This study has demonstrated that both class I and II HLA genotypes have a role in amoxicillin/clavulanate DILI susceptibility, thus indicating the significance of the adaptive immune response in the pathogenesis of DILI. Although the HLA genotypes which have been identified may be useful for future studies into the pathogenesis of DILI, their low positive predictive value suggests they have limited utility as a predictive biomarker of amoxicillin/clavulanate DILI; however, their high negative predictive value may have a role in DILI diagnosis.

Although the precise pathophysiological mechanisms underlying the phenotype-genotype association in amoxicillin/clavulanate-induced DILI is still unknown, it is thought to be immunological due to specific HLA subtype involvement. HLA-DR is a major histocompatibility complex (MHC) class II cell surface receptor which is encoded by the HLA complex on a section of chromosome 6. These HLA-DR receptor and its ligand, which are typically a peptide consisting of nine amino acids or more, constitutes a ligand for T cell receptors. It is thought that either drug or drug-class specific factors have a role in the initiating event of DILI which constitutes the reactive metabolite covalently binding to a protein to form an adduct. The antigen-presenting cells process this hapten; it undergoes cleavage to form peptide fragments which are presented to T cells through MHC class I or II molecules and co-stimulatory signals. This, together with the properties of the cytokine micro-environment within the liver, determines both the nature and magnitude of the DILI response [4].

The average onset of symptoms from first starting amoxicillin/clavulanate is 18 days. In these patients, there is often the presence of features associated with hypersensitivity including rash, fever and eosinophilia. The majority of patients make a full recovery within 4–6 months; however, there are a select few patients who go on to develop fulminant hepatic failure and require transplantation. Fortunately, this is rare.

Research has not decisively established that these ‘risk alleles’ are truly causative of DILI; studies have merely reported very strong associations between them both. There is a need to understand the pathophysiology of amoxicillin/clavulanate DILI in order to explain why only a small proportion of patients with these specific genetic variations develop this complication, to avoid denying this important antimicrobial to a large number of patients. Further studies into these areas of uncertainty and the direct role of the immune system in DILI are required before elucidating the impact of genetic screening prior to amoxicillin/clavulanate treatment.

## Flucloxacillin-Associated DILI

Flucloxacillin is used for the treatment of staphylococcal infections and has been shown to precipitate, typically the cholestatic form, of DILI. In the UK, flucloxacillin is the second commonest cause of drug-induced jaundice [17] despite the incidence of flucloxacillin DILI being low, reported as 8.5 cases per 100,000 new users [29]. Flucloxacillin accounts for 16% of all DILI cases in Sweden, where it is the most common cause of idiosyncratic liver disease [30].

A seminal GWAS study which analysed 51 patients with flucloxacillin-induced DILI reported a strong association with HLA-B*57:01 (OR = 80.6; 95% CI = 22.8–284.9); the presence of this allele was associated with an 81-fold increased risk of DILI secondary to flucloxacillin. In a replication cohort, this association was still significant with the results suggesting that if an individual tested positive for HLAB*57:01, they would have a 100-fold increased risk of developing DILI with flucloxacillin use [31, 32]. The absolute risk for this reaction was calculated as 1:500–1:1000. This discovery is particularly interesting as it is the same HLA allele which is associated with abacavir sensitivity, as previously discussed, where hepatic injury is not a typical feature. The underlying pathogenesis of flucloxacillin-induced DILI has been described by Monshi et al. [31]. Flucloxacillin has been shown to covalently bind to select lysine residues on circulating albumin, the degree of binding controls, the intensity of the T cell proliferative response and subsequent immune reaction. In addition, flucloxacillin-specific mononuclear cell responses were found in peripheral blood samples taken from individuals with a history of DILI. One study characterised flucloxacillin reactive CD4+ and CD8+ T cell clones from samples from these individuals, whilst another study has shown flucloxacillin’s ability to activate naïve CD8+ T cells from HLA-B*5701 positive volunteers [33].

With regards to pretreatment testing of HLAB* 57:01, the sensitivity and specificity was high at 87 and 94%, respectively. However, due to the low incidence of flucloxacillin associated DILI, the positive predictive value of the test would be a mere 0.12%. In translating these figures to clinical practice, more than 13,500 patients would need to be tested before 1 case of flucloxacillin DILI could be avoided [34]. Also, in doing this many patients who test HLAB* 57:01 positive who would not have developed DILI with flucloxacillin would be denied this effective therapy. It was therefore thought that isolated routine HLAB* 57:01 screening prior to flucloxacillin use would be neither economically nor clinically viable for DILI prevention.

## Minocycline-Associated DILI

Minocycline is a semi-synthetic tetracycline derivative and bacteriostatic antibiotic. It is mainly effective against coagulase-negative staphylococci and Propionibacterium acnes; it’s most common indication is in the treatment of acne vulgaris [35]. For this indication, minocycline is given for a prolonged period, this can be months to years, and the treatment cohort is typically young patients who are otherwise healthy.

Gough et al. described the association between minocycline and DILI in 1996 after investigating a number of cases which had reported to the Committee on Safety of Medicines [36]. Since then, a number of patient studies have supported this association [37–39]. This research also reports characteristic clinical features such as systemic arthralgia and detectable autoantibodies particularly in young women [37].

Urban et al. conducted a GWAS study in 25 Caucasian patients with minocycline-associated DILI comparing their genotype to an unexposed population control. Within the DILI patient cohort, 80% were female, the median age was 19 years and median latency from minocycline initiation and DILI onset was 318 days. The indication for minocycline use in all cases was dermatological. This study reported a significant association between HLA-B*35:02 and risk of minocycline-DILI with a 16% carrier frequency in DILI cases compared with a 0.6% in the population control (odds ratio 29.6, 95% CI: 7.8–89.8, *p* = 2.5 × 10^−8^). The GWAS results were confirmed with sequence-based HLA typing. Studies using in silico modelling techniques have supported the hypothesis that the direct binding of minocycline to this novel HLA allele may have an important role in the initiation of minocycline-DILI [40•]. The authors propose a number of theories regarding the mechanism of minocycline-induced liver injury; however, the link between HLA-B*35:02 and minocycline is still unclear.

Within the general population the HLA-B*35:02 allele has a low frequency of only 0.3% in Caucasians and less than 0.1% in African Americans. Given that the incidence of minocycline-associated DILI is so low, pre-treatment HLA typing is unlikely to prevent this condition. However, genotyping for HLA-B*35:02 may be helpful as a diagnostic aid in patients with a suspected minocycline-associated DILI to distinguish it from autoimmune hepatitis.

## Other Examples of Genetic Associations and DILI

A number of other studies have identified genetic risk factors linking specific drugs to DILI. Both HLA-DR and HLA-DQ genotypes were found to be predictive for DILI induced by lumiracoxib (HLA-DRB1*1501-HLA-DQB1*0602-HLA-DRB5*0101-HLA-DQA1*0102, most significant allele *P* = 6.8 × 10–25 allelic, odds ratio = 5.0, 95% CI 3.6–7.0) [9]. This selective cyclooxygenase-2 inhibitor has been recently removed from the market due to hepatotoxicity [9, 10]. Carriers of the HLA allele DQA*02:01 have been associated with lapatinib-induced DILI, a tyrosine kinase inhibitor used in the treatment of breast cancer. (*P* < .001, odds ratio = 9.0, 95% CI 3.2–27.4) [41]. An overview of the most important HLA associations uncovered by GWAS is presented in Table 1.

**Table 1:** Overview of HLA associations with DILI

Drug	HLA Allele	Type of study	Approximate odds ratio	Reference(s)
Amoxicillin-clavulanate	DRB1*15:01	GWAS and candidate gene	3	
	DQB1*06:02			[22, 23, 28]
	A*02:01		2.2	
	B*18:01		4.0	
Flucloxacillin	B*57:01	GWAS	80	[31, 32]
Minocycline	B*35:02	GWAS	29.6	[40•]
Lumiracoxib	DRB1*15:01	GWAS	5	[9]
	DQB1*06:02			
Lapatinib	DQA*02:01	GWAS and candidate gene	9	[41]
Ximelagatran	DRB1*07:01	GWAS	Not available (*P* = 6.0 × 10^−6^)	[43]
	DQA1*02:01			
Ticlopidine	A*33:03	Candidate gene	13	[44]

*GWAS* genome-wide association studies

The most recent research conducted by the international DILI Consortium was a GWAS looking into genetic risk factors for DILI caused by licenced drugs which do not have any previously reported genetic associations. GWAS was performed on 862 patients with DILI and 10,588 population matched controls. All patients were of European ancestry, and their DILI was associated with one particular drug, patients with amoxicillin/clavulanate or flucloxacillin DILI were not included in this study. DILI was associated with the single nucleotide polymorphism (SNP) *rs114577328* (a proxy for A*33:01, a HLA class 1 allele; odds ratio [OR] 2.7 with a 95% confidence interval [CI] of 1.9–3.8, *P* = 2.4 × 10^−8^) [42•]. Terbinafine, fenofibrate and ticlopidine-related DILI was shown to have an association with this A*33:01 genotype. Interestingly, this novel class 1 allele is relatively rare amongst the general population. Further phenotypical analysis indicated that A*33:01-associated DILI was typically a cholestatic or mixed form of DILI, not hepatocellular. Another genetic association made through this GWAS study was the SNP *rs72631567* on chromosome 2 (OR 2, 95% CI of 1.6–2.5, *P* = 9.7 × 10^−9^) [42•]. This variant was present in a number of patients with DILI from a variety of drugs and gave a range of cholestatic, mixed and hepatocellular forms of DILI.

Again, as with the other HLA associations and DILI, the overall sensitivity and specificity of the A*33:01 allele as a predictor of this adverse reaction is low. However, this association may be significant in the future treatment of patients with DILI associated with the A*33:01 allele.

## Conclusion

In order to determine the benefit of a pretreatment genetic screening test and therefore whether there is scope for it in clinical practice, it is useful to quantify the change in the incidence of DILI in a given proportion of people with the associated genetic susceptibility marker. The attributable proportion (AP) can be used to calculate this and has been reported in a select number of the current studies such as the amoxicillin/clavulanate study by Donaldson et al. . [8]. This value represents the proportion of disease-cases attributable to the biomarker and is based on the sensitivity of the laboratory testing as well as the relative risk of association between the biomarker and disease established from case control, cohort studies or by clinical trial. This type of analysis would be useful in this setting given the complex nature of genetic studies; not all patients with the gene or single nucleotide polymorphism develop the disease and conversely not all patients with the disease have the associated genetics. Although this point implies that not everyone who will suffer DILI will be revealed through the use of this biomarker, it is important to focus on improving the outcome for the patients who are highlighted through the screening since this is what the research has shown- their increased susceptibility.

With the exception of abacavir, there is currently no other prospective genetic screening tests which have met the threshold for clinical application; generally, the incidence of DILI is too low to warrant the cost and effort associated with screening. However with the development of the concept of personalised medicine, potentially, a large panel of genes for disease susceptibility, drug efficacy and adverse drug reactions could be check once in a lifetime, thereby completely changing the cost-effectiveness paradigm. This panel of genes could allow medical professionals to make personalised drug and treatment choices and, with reference to this paper, to highlight which medications should be avoided in patients with specific HLA alleles to reduce the risk of DILI.

Although the research does not support the use of genetic testing for prevention of DILI, there is evidence to support its use in diagnosis. The majority of HLA alleles associated with DILI have a high negative predictive value (NPV). An example of this, which has been previously mentioned, is in Lucena et al.’s study in 2011 on amoxicillin/clavulanate DILI. The researchers reported the best NPV, expressed as 1-NPV, in two different ethnic cohorts as 0.006 and 0.007% based on the absence of A*0201 and rs2523822/C, respectively [28]. Genotyping could therefore be used to rule out adverse hepatic reactions in patients on particular drugs thus allowing the consideration of alternative diagnoses. The reliability of the negative predictive value of genetic testing can also be used to identify the correct medication underlying the DILI in a situation where the patient has been exposed to two concomitant drugs.
